# Characterization of Three Complete Mitochondrial Genomes of *Hemiculter* and Comparative Mitogenomic Analysis of Cultrinae Fishes

**DOI:** 10.3390/ijms27146325

**Published:** 2026-07-16

**Authors:** Seung Hyun Lee, Sang Ki Kim, Seung Woo Jang, Kyong In Suh, Jong Kyun Park, Kang-Rae Kim

**Affiliations:** 1 Diversity Conservation Department, Nakdonggang National Institute of Biological Resources, Sangju 37242, Republic of Korea; shlee86@nnibr.re.kr (S.H.L.); ivoice@nnibr.re.kr (S.K.K.); seungwoojang@gmail.com (S.W.J.);; 2 Department of Ecological Science, Kyungbuk National University, Sangju 37224, Republic of Korea; entopark@knu.ac.kr; 3 Southeast Sea Fisheries Research Institute, National Institute of Fisheries Science, Namhae 52440, Republic of Korea

**Keywords:** mitogenome, *Hemiculter leucisculus*, *Hemiculter eigenmanni*, Cultrinae

## Abstract

The genus *Hemiculter* includes closely related freshwater fishes widely distributed across East Asia, and several species are difficult to distinguish using external morphology alone. Mitochondrial markers have been widely used for species identification and phylogenetic inference in fishes, but single-gene approaches may be insufficient when nominal species show recent divergence, mitochondrial introgression, or taxonomic inconsistency. In this study, we newly assembled and characterized three complete mitogenomes of *Hemiculter*: *H. leucisculus* from Korea, *H. leucisculus* from Mongolia, and *H. eigenmanni* from Korea. The three mitogenomes contained the typical 37 mitochondrial genes and showed conserved genome organization, base composition, codon usage, and tRNA structures. Comparative analysis of concatenated 13 protein-coding genes revealed uneven sequence variability, with relatively high nucleotide diversity in *ND2*, *ND1*, *ND6*, and *ND5*. Pairwise K2P distances and phylogenetic analyses showed that the Korean and Mongolian *H. leucisculus* mitogenomes formed a distinct clade, evidencing that sequences assigned *to H. leucisculus* did not form a single consensus mitochondrial lineage. Barcode-gap overlap appeared to reflect discordance between current species assignments and mitogenomic relationships rather than the absence of informative barcode genes. These results indicate that *Hemiculter* mitogenomes are structurally conserved but informative for detecting lineage-level divergence and taxonomic inconsistency. Our findings suggest that the *H. leucisculus*–*H. eigenmanni* complex requires further integrative reassessment using broader sampling, voucher-based morphology, and nuclear genomic evidence.

## 1. Introduction

The genus *Hemiculter* is a group of small cyprinid fishes widely distributed in East Asian freshwater systems, including Korea, China, Japan, Hong Kong, and the Amur River basin [1,2]. In the Korean Peninsula, *Hemiculter* includes several closely related species, including *H. leucisculus*, *H. bleekeri*, and *H. eigenmanni* [2,3]. These species are ecologically similar and often occur in riverine and reservoir habitats, where they occupy upper-water habitats and feed on aquatic insects, algae, and other food resources [4,5]. Although adult individuals can be distinguished morphologically, juveniles and closely related congeners are often difficult to identify reliably based only on external characters [6,7]. This difficulty is particularly important for biodiversity assessment, faunal surveys, and taxonomic studies of closely related freshwater fishes [6,7,8,9].

Among Korean *Hemiculter*, the taxonomic status of *H. eigenmanni* has been particularly problematic [2,10]. Although *H. eigenmanni* has been treated in some Korean faunal and taxonomic contexts, previous morphological work proposed that *H. eigenmanni* should be regarded as a junior synonym of *H. leucisculus* [2,10]. Subsequent mitochondrial *CYTB* analysis also showed a close relationship between Korean *H. eigenmanni* and Chinese *H. leucisculus*, but the relationships among Korean *Hemiculter* taxa were not fully resolved and required further examination using type specimens and broader comparative material [2].

Mitochondrial DNA has been widely used for species identification and phylogenetic inference because of its high copy number, maternal inheritance, relatively rapid substitution rate, and conserved genomic organization [11,12]. Single mitochondrial markers such as cytochrome c oxidase subunit I (*COI*) and cytochrome b (*CYTB*) have been widely applied in fish identification and molecular taxonomy [8,13]. However, single mitochondrial genes do not always provide sufficient resolution for closely related, recently diverged, or sympatric species [9,14]. In some freshwater fishes, mitochondrial markers may show overlapping intra- and interspecific distances, incomplete lineage sorting, introgression, or discordance with morphological taxonomy [9,15]. Therefore, reliance on a single mitochondrial marker may obscure taxonomic complexity within closely related species groups [15,16].

Complete mitochondrial genomes can provide more informative sequence data than single-gene markers because they include 13 protein-coding genes (PCGs), two rRNA genes, 22 tRNA genes, and the control region [11]. Comparative mitogenomic analyses allow simultaneous evaluation of genome organization, nucleotide composition, codon usage, tRNA structure, sequence variability, phylogenetic relationships, and selection pressure [17,18]. In addition, concatenated mitochondrial PCGs can improve phylogenetic resolution and provide quantitative evidence for lineage divergence, especially in groups where single markers show limited discriminatory power [19,20]. Thus, complete mitogenomes are useful resources for clarifying relationships among closely related fish taxa and for detecting potential inconsistencies in public sequence records [11,20,21].

This issue is relevant to *Hemiculter* because previous studies based on partial mitochondrial markers have suggested that *H. leucisculus* sensu lato may include geographically structured mitochondrial lineages, and that molecular results should be interpreted together with voucher-based morphology and broader geographic sampling [22,23]. However, most previous studies have relied on partial mitochondrial markers, and complete mitogenome-scale comparisons remain limited for evaluating whether currently available *Hemiculter* records form taxonomically consistent mitochondrial lineages [23].

Previous studies have examined the molecular phylogeny and taxonomic status of *Hemiculter* using partial mitochondrial markers and morphological evidence [2,24]. However, comparative mitogenomic information remains limited for *Hemiculter*, and the extent to which complete mitogenomes can resolve relationships among closely related *Hemiculter* lineages has not been fully evaluated [25,26]. In particular, because public databases contain mitogenomes assigned to several *Hemiculter* species, comparative analysis with newly generated mitogenomes provides an opportunity to test whether current taxonomic labels are consistent with whole-mitogenome phylogenetic and distance-based evidence [22,23].

Because *H. leucisculus* is widely distributed across East Asia, including the Korean Peninsula, China, Mongolia, and the Amur River basin, mitogenomic data from geographically distinct regions are important for evaluating regional mitochondrial variation and the consistency of currently available *Hemiculter* records [9,23,27]. Korean and Mongolian samples represent geographically separated regional samples within this broad distributional context; therefore, adding complete mitogenomes from these regions can improve the regional representation of currently available comparative datasets [28]. Therefore, the present sampling was designed to add complete mitogenomic records from underrepresented regional samples and to evaluate their phylogenetic placement within the available Cultrinae mitogenomic framework, rather than to infer population-level phylogeographic patterns across the entire distribution of *H. leucisculus*.

Accordingly, the central question of this study was not to formally revise the taxonomy of *Hemiculter*, but to test whether newly generated complete mitogenomes are phylogenetically consistent with currently available public *Hemiculter* records [2]. This is especially important for evaluating whether *H. leucisculus*, including geographically distinct Korean and Mongolian samples and public accessions, forms a single consensus mitochondrial lineage, and whether the mitochondrial placement of *H. eigenmanni* supports or complicates the previously proposed synonymy with *H. leucisculus* [2,27].

In this study, we newly assembled and characterized three complete mitochondrial genomes of *Hemiculter*: *H. leucisculus* from Korea, *H. leucisculus* from Mongolia, and *H. eigenmanni* from Korea. We compared these mitogenomes with publicly available *Hemiculter* and Cultrinae mitogenomes to examine genome structure, sequence variability, pairwise genetic distances, barcode-gap patterns, phylogenetic relationships, and selection pressure. By doing so, we aimed to assess the utility of mitogenome-scale data for detecting lineage-level divergence and taxonomic inconsistency among closely related *Hemiculter* lineages.

## 2. Results

### 2.1. Mitochondrial Genome Organization and Base Composition

The total mitochondrial genome lengths of *H. leucisculus* from Korea, *H. leucisculus* from Mongolia, and *H. eigenmanni* from Korea were 16,623, 16,618, and 16,745 bp, respectively (Table 1). The three newly sequenced voucher specimens generated 11.46–15.91 Gb of raw data, with filtered Q30 values ranging from 93.22% to 93.99%, and their detailed sequencing statistics are presented in Table S1. Each mitogenome contained the typical set of 37 mitochondrial genes, including 13 PCGs, two rRNAs, and 22 tRNAs, together with the major non-coding control region and a putative origin of L-strand replication region (Table S2). AT-skew and GC-skew values were calculated for the PCGs of the three mitogenomes (Table S2). AT-skew values varied among PCGs, with most genes showing positive values, whereas *COX1*, *ND3*, *ATP6*, and *ND6* showed negative values in one or more of the three mitogenomes. GC-skew values were negative for all PCGs except *ND6*, indicating strand-specific nucleotide compositional bias (Table S2). *ND5* used different complete stop codons among the three mitogenomes, with TAA in *H. leucisculus* from Korea and *H. leucisculus* from Mongolia and TAG in *H. eigenmanni* from Korea. All PCGs showed the typical strand orientation observed in fish mitogenomes, with *ND6* encoded on the opposite strand relative to the other PCGs. Most tRNA genes were also encoded on the negative strand, whereas trnE, trnP, trnQ, trnA, trnN, trnC, trnY, and trnS2 were encoded on the positive strand.

### 2.2. Sequence Variability of Mitochondrial PCGs

**Figure 1:**
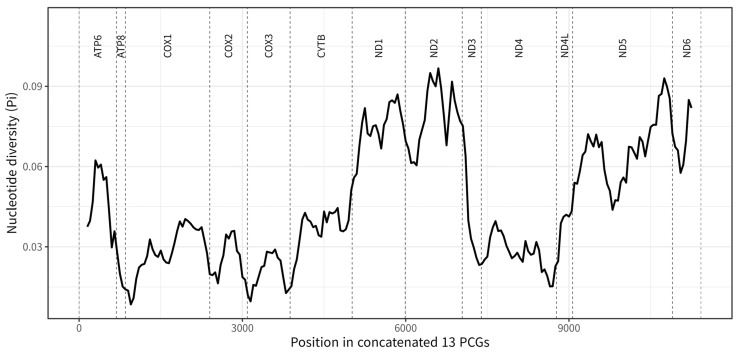
Sliding-window analysis of nucleotide diversity across concatenated 13 mitochondrial protein-coding genes. Nucleotide diversity (Pi) was calculated using a 300 bp window and a 50 bp step size. Dashed vertical lines indicate gene boundaries, and gene names indicate the concatenation order of the 13 PCGs.

Sequence variability differed among the 13 mitochondrial protein-coding genes of *Hemiculter* mitogenomes (Table 2). *ND2* showed the highest nucleotide diversity (Pi = 0.08047), followed by *ND1* (Pi = 0.07846), *ND6* (Pi = 0.07079), and *ND5* (Pi = 0.06747). In contrast, *COX3* showed the lowest nucleotide diversity (Pi = 0.02009), followed by *ATP8* (Pi = 0.02112) and *COX2* (Pi = 0.02437). *ND5* contained the largest number of variable sites (393) and parsimony-informative sites (245), whereas *ATP8* contained the fewest variable sites (9). Overall, NADH dehydrogenase genes showed relatively high sequence variability, while COX genes and *ATP8* were comparatively conserved (Table 2; Figure 1).

### 2.3. Pairwise Genetic Distance Among Hemiculter Mitogenomes

Pairwise K2P distances based on concatenated 13 mitochondrial PCGs showed clear genetic structuring among *Hemiculter* mitogenomes (Table 3). The newly assembled Korean and Mongolian *H. leucisculus* mitogenomes were very closely related to each other (PZ324712–PZ324711: K2P = 0.002809). In contrast, these two mitogenomes were highly divergent from the public *H. leucisculus* NC_022929 (K2P = 0.053518–0.054192) and from AP012110 and MZ520999 (K2P = 0.080043–0.081365). The lowest K2P distance was observed between *H. leucisculus* KF956522 and *H. tchangi* NC_036740 (K2P = 0.001403), followed by *H. leucisculus* NC_022929 and *H. eigenmanni* NC_029388 (K2P = 0.001491). The newly assembled *H. eigenmanni* PZ324713 was closer to *H. leucisculus* NC_022929 and *H. eigenmanni* NC_029388 than to the newly assembled *H. leucisculus* mitogenomes. In addition, *H. bleekeri* KT361083 was closer to *H. tchangi* NC_036740 and *H. leucisculus* KF956522 (K2P = 0.006158) than to *H. bleekeri* NC_029831 (K2P = 0.089341). These results indicate that several public *Hemiculter* records show mitochondrial discordance with their current species names (Table 3).

### 2.4. Control-Region Length and Sequence Variation

The mitochondrial control-region alignment had a uniform aligned length of 937 bp across all analyzed *Hemiculter* mitogenomes (Table S3). Ungapped lengths showed only limited variation among sequences, ranging from 927 to 937 bp. The shortest control region was observed in *H. bleekeri* NC_029831, whereas several sequences, including *H. leucisculus* MZ521000 and *H. eigenmanni* NC_029388, showed the longest ungapped lengths.

### 2.5. PCG Features and Codon Usage Patterns

The three newly assembled *Hemiculter* mitogenomes contained 13 mitochondrial PCGs, with lengths ranging from 165 bp for *ATP8* to 1836 bp for *ND5*. Twelve PCGs used ATG as the start codon, whereas *COX1* used GTG in all three newly assembled mitogenomes. Stop codons included the complete stop codons TAA and TAG, as well as truncated stop codons such as T-- and TA-. These start and stop codon patterns were generally consistent among the three newly assembled mitogenomes.

**Figure 2:**
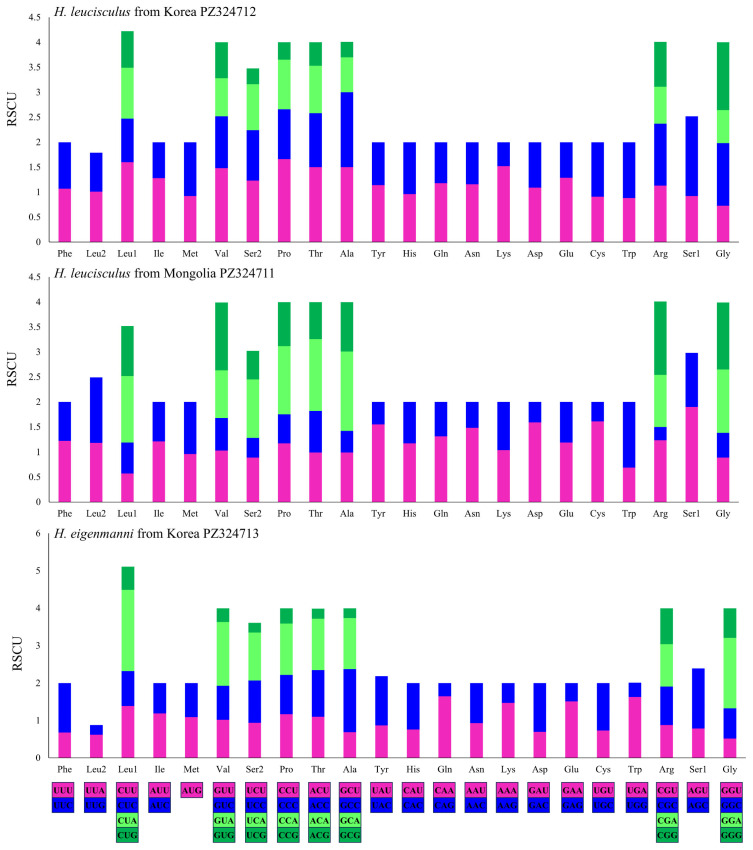
Relative synonymous codon usage (RSCU) summary of the three newly assembled *Hemiculter* mitogenomes.

Codon usage patterns were broadly similar among the three newly assembled *Hemiculter* mitogenomes (Figure 2). The total codon numbers were 3808 in *H. leucisculus* from Korea, 3807 in *H. leucisculus* from Mongolia, and 3808 in *H. eigenmanni* from Korea. Codon usage patterns were broadly similar among the three newly assembled *Hemiculter* mitogenomes (Figure 2). Overall, codon usage was biased toward A/T-ending codons, consistent with the AT-rich composition of mitochondrial PCGs.

**Figure 3:**
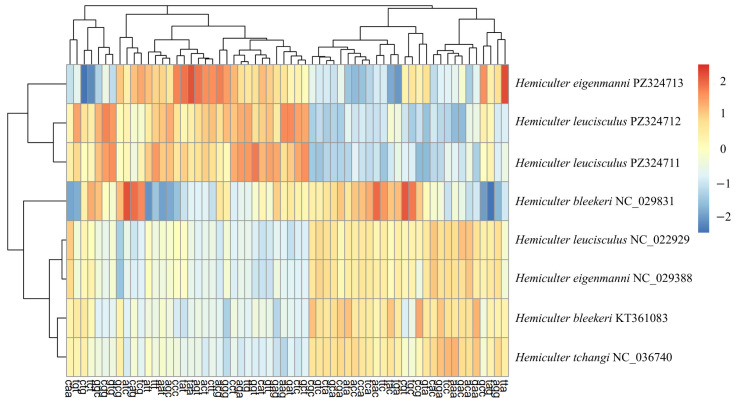
Relative synonymous codon usage (RSCU) heatmap based on concatenated 13 mitochondrial protein-coding genes.

The RSCU heatmap showed broadly conserved codon usage patterns among the analyzed *Hemiculter* mitogenomes (Figure 3). *H. bleekeri* NC_029831 showed a distinct codon usage profile compared with other *Hemiculter* sequences, particularly in several codons with high relative usage values. The newly assembled *H. leucisculus* PZ324711 and PZ324712 clustered closely together, indicating similar codon usage patterns between the Mongolian and Korean samples. In contrast, public *H. leucisculus* accessions did not form a single uniform pattern, suggesting codon usage heterogeneity among sequences assigned to the same species.

### 2.6. Barcode-Gap Analysis of Individual Mitochondrial PCGs

**Figure 4:**
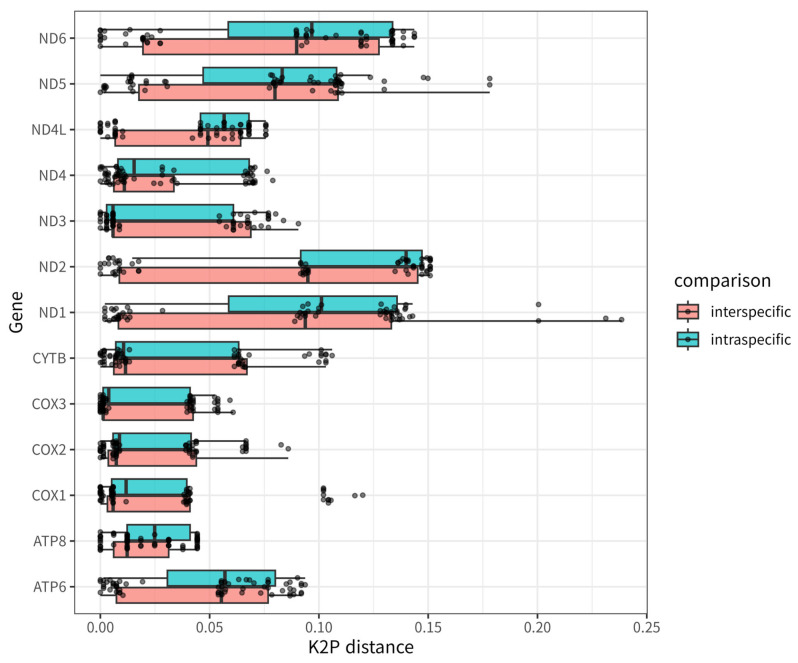
Barcode-gap analysis based on individual mitochondrial protein-coding genes.

Barcode-gap analysis revealed substantial overlap between intra- and interspecific K2P distances across most mitochondrial PCGs (Figure 4). *ND1*, *ND2*, *ND5*, and *ND6* showed relatively high divergence, but elevated intraspecific distances were also observed. *COX1* and *CYTB* likewise did not show a clear separation between intra- and interspecific variation. This overlap was mainly associated with discordant public records assigned to the same nominal species, rather than with uniformly low discriminatory power of mitochondrial genes.

### 2.7. Transfer RNAs 

**Figure 5:**
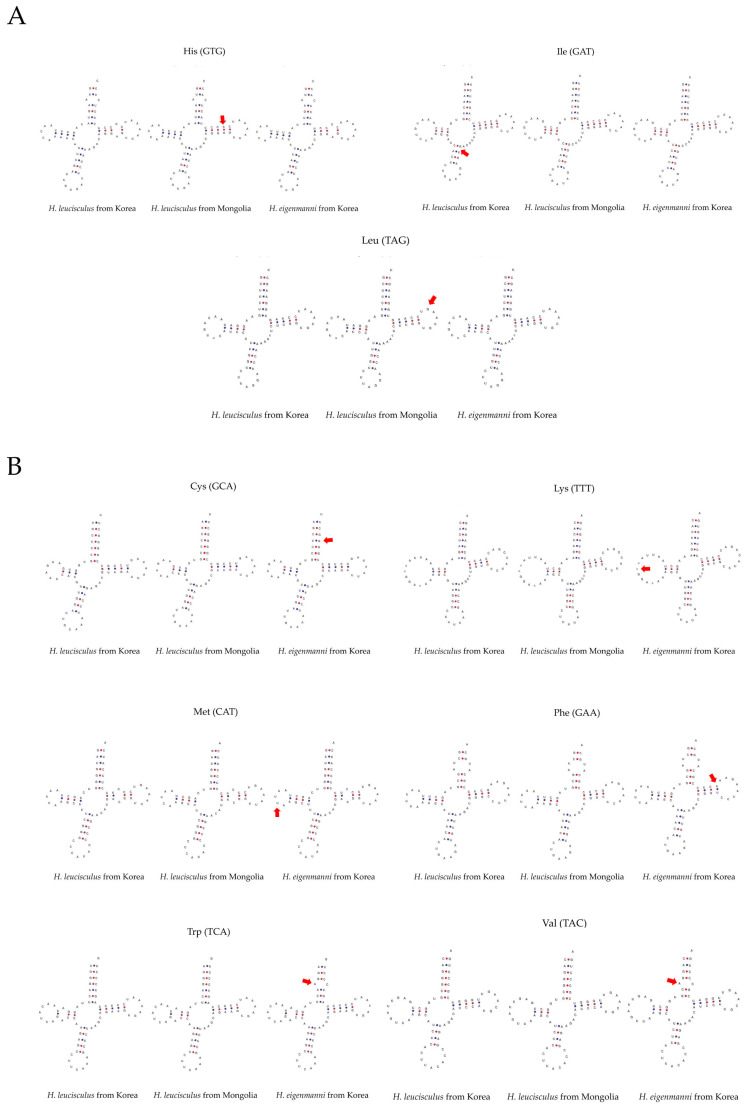
Predicted tRNA secondary structures of the three newly assembled *Hemiculter* mitogenomes. (**A**) tRNA secondary structure differences between *H. leucisculus* from Korea and *H. leucisculus* from Mongolia. (**B**) tRNA secondary structure differences between *H. leucisculus* and *H. eigenmanni*. Red arrows indicate differing sites.

Each of the three newly assembled *Hemiculter* mitogenomes encoded 22 tRNA genes, ranging in size from 68 to 76 bp (Table 1). The three mitogenomes showed generally conserved tRNA gene lengths and predicted secondary structures, although minor sequence and structural differences were detected in several tRNAs (Figure 5). Differences in predicted tRNA secondary structures between *H. leucisculus* from Korea and *H. leucisculus* from Mongolia were observed in tRNA-His, tRNA-Ile, and tRNA-Leu. Differences between *H. leucisculus* and *H. eigenmanni* were observed in tRNA-Cys, tRNA-Lys, tRNA-Met, tRNA-Phe, tRNA-Trp, and tRNA-Val.

### 2.8. Phylogenetic Analysis

**Figure 6:**
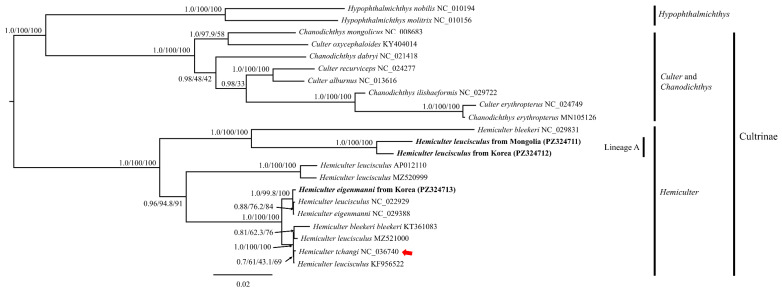
Phylogenetic tree inferred from concatenated 13 mitochondrial protein-coding genes using partitioned maximum likelihood analysis in IQ-TREE2. The concatenated 13-PCG alignment was partitioned by gene, and the best-fitting substitution models and partitioning scheme were selected using ModelFinder implemented in IQ-TREE2. Node values indicate BI posterior probability and SH-aLRT support values and ultrafast bootstrap support values, respectively. *Hypophthalmichthys nobilis* NC_010194 was designated as the outgroup species for rooting the tree. The newly sequenced *Hemiculter* mitogenomes are shown in bold. The red arrows indicate species that are highlighted due to a mismatch in the phylogenetic tree.

Phylogenetic reconstruction based on concatenated 13 mitochondrial PCGs resolved *Hemiculter* as a distinct lineage within Cultrinae (Figure 6). The newly assembled *H. leucisculus* mitogenomes from Korea and Mongolia formed a well-supported clade, indicating close mitogenomic affinity between the two samples. The newly assembled *H. eigenmanni* PZ324713 was recovered in close association with *H. eigenmanni* NC_029388, supporting its mitochondrial placement within the *H. eigenmanni*-related lineage. However, several public *Hemiculter* mitogenomes showed incongruence between their nominal species assignments and phylogenetic positions. Sequences assigned to *H. leucisculus* were not recovered as a monophyletic group; instead, *H. leucisculus* NC_022929 clustered closer to the *H. eigenmanni* lineage than to the newly assembled *H. leucisculus* clade. *H. tchangi* NC_036740 showed close mitogenomic affinity with public *H. leucisculus* records, including KF956522 and MZ521000.

### 2.9. dN/dS Analysis of Mitochondrial PCGs

**Figure 7:**
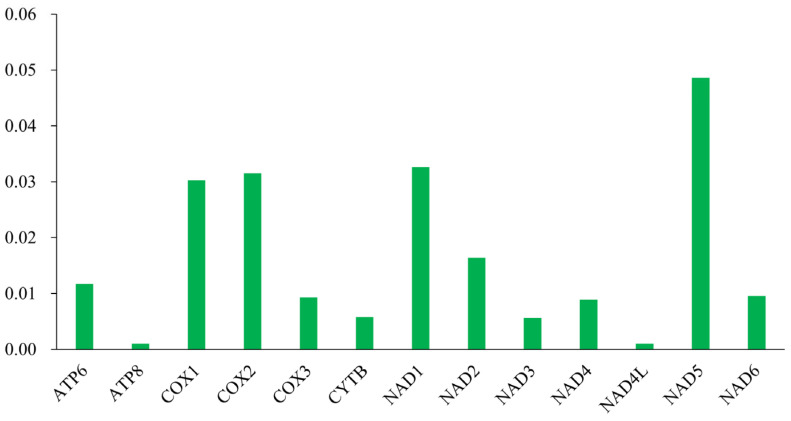
Nonsynonymous/synonymous substitution ratios (dN/dS, ω) of 13 mitochondrial protein-coding genes from 20 Cultrinae mitogenomes.

The dN/dS ratios (ω) of all 13 mitochondrial PCGs were lower than 1, indicating that these genes are under purifying selection in Cultrinae mitogenomes (Figure 7). Among them, *ND5* showed the highest ω value, followed by NAD1, *COX2*, and *COX1*, suggesting relatively higher nonsynonymous substitution rates in these genes. In contrast, *ATP8* and NAD4L showed the lowest ω values, indicating stronger functional constraint.

## 3. Discussion

In this study, we characterized three complete mitochondrial genomes of *Hemiculter* and compared them with publicly available mitogenomes of *Hemiculter* and related Cultrinae fishes. The three newly assembled mitogenomes, representing *H. leucisculus* from Korea, *H. leucisculus* from Mongolia, and *H. eigenmanni* from Korea, showed the typical vertebrate mitochondrial genome organization, including 13 protein-coding genes (PCGs), 22 tRNA genes, and two rRNA genes. Their genome sizes, gene order, nucleotide composition, start and stop codon usage, and tRNA organization were broadly conserved. This pattern is consistent with the general structure of teleost mitochondrial genomes, which are typically compact, maternally inherited, and composed of a conserved set of coding and non-coding regions [17,29,30]. Therefore, the mitogenomic differences observed among the analyzed *Hemiculter* lineages appear to be mainly associated with nucleotide substitutions, codon usage variation, control-region sequence divergence, and lineage-specific phylogenetic placement rather than major changes in gene content or gene order [17,31].

The PCG features of the three newly assembled mitogenomes were generally consistent with those reported in other fish mitogenomes [32,33]. Most PCGs used ATG as the start codon, whereas *COX1* used GTG in all three newly assembled *Hemiculter* mitogenomes. Incomplete stop codons were also detected in several PCGs, including *COX2*, *CYTB*, and *ND4*. These features are commonly reported in vertebrate mitogenomes and are generally interpreted as typical mitochondrial codon usage patterns rather than lineage-specific functional changes [31,33]. Thus, the start and stop codon patterns observed in this study should be interpreted as typical mitochondrial genomic features rather than as evidence of lineage-specific functional divergence [31,32].

Although the overall mitogenomic structure was conserved, the 13-PCG dataset revealed clear heterogeneity in sequence variability among mitochondrial genes [17,34]. Gene-wise nucleotide diversity showed that *ND2* had the highest Pi value, followed by *ND1*, *ND6*, and *ND5*, whereas *COX3* showed the lowest Pi value, followed by *ATP8* and *COX2*. *ND5* also contained the largest number of variable sites, while *ATP8* contained the fewest. The sliding-window analysis further showed that nucleotide diversity was not evenly distributed across the concatenated 13-PCG alignment but was concentrated in particular PCG regions. These results suggest that several mitochondrial genes, especially NADH dehydrogenase genes, contain relatively more nucleotide-level variation useful for evaluating relationships among *Hemiculter* lineages [34,35]. However, higher nucleotide diversity does not automatically mean that a gene is a better species-identification marker [9,16]. A useful barcode marker should show low intraspecific variation, high interspecific divergence, and a clear separation between intra- and interspecific distances [13,36]. Therefore, Pi values should be interpreted together with pairwise genetic distances and barcode-gap patterns [16].

The pairwise distance and phylogenetic analyses revealed that the taxonomic inconsistency within *Hemiculter* is not limited to a single discordant accession but reflects a broader lack of topological consensus among sequences assigned to *H. leucisculus*, *H. eigenmanni*, *H. bleekeri*, and *H. tchangi* [2,23,26]. Previous taxonomic studies have treated *H. eigenmanni* as a junior synonym of *H. leucisculus* based on morphological comparisons of type and Korean specimens [10], and mitochondrial *CYTB* analysis also suggested that Korean *H. eigenmanni* is closely related to Chinese *H. leucisculus* [2]. However, the present mitogenomic tree does not simply provide unambiguous support for synonymizing *H. eigenmanni* with *H. leucisculus*. Instead, the newly assembled Korean and Mongolian *H. leucisculus* mitogenomes formed a separate, well-supported clade, whereas some public sequences assigned to *H. leucisculus* were recovered in different positions, including an *H. eigenmanni*-related clade and a clade close to *H. tchangi*. Thus, sequences currently labeled as *H. leucisculus* did not form a single consensus mitochondrial lineage. This phylogenetic pattern should be interpreted in the context of molecular identification studies showing that mitochondrial sequence-based clades are most informative for species-level discrimination when they are compared with reliable reference haplotypes and voucher-based morphological evidence [37,38].

This topology is taxonomically important because the close placement of some public *H. leucisculus* accessions with *H. eigenmanni*-related sequences could be interpreted as being consistent with the previously proposed synonymy [2,10]. However, the overall tree structure does not provide a stable basis for applying that synonymy without further evidence. If *H. eigenmanni* were simply synonymous with *H. leucisculus*, *H. eigenmanni*-related sequences and *H. leucisculus* accessions would be expected to form a coherent and taxonomically interpretable mitochondrial lineage. In contrast, the present tree shows that the newly assembled *H. leucisculus* from Korea and Mongolia are separated from the *H. eigenmanni*-related group by distinct branch lengths, whereas other public *H. leucisculus* records are scattered across different *Hemiculter* lineages. Therefore, the present result should be interpreted as evidence of taxonomic controversy within the *H. leucisculus*–*H. eigenmanni* complex rather than as definitive support for synonymy.

The lack of a consensus mitochondrial topology among *H. leucisculus* accessions also suggests that the current taxonomic labels of public *Hemiculter* mitogenomes should be treated cautiously [23,26,28]. The discordant placement of *H. leucisculus* NC_022929 near the *H. eigenmanni*-related lineage, the separate placement of the newly assembled Korean and Mongolian *H. leucisculus* clade, and the close affinity of *H. leucisculus* KF956522 with *H. tchangi* NC_036740 collectively indicate that the public database may include misidentified records, geographically structured lineages, unresolved synonymy, incomplete taxonomic resolution, mitochondrial introgression, or lineage-level divergence within nominal species [15,39]. This interpretation is consistent with previous studies showing that *H. leucisculus* sensu lato contains multiple mitochondrial or phylogeographic lineages and that molecular results in *Hemiculter* should be interpreted together with voucher-based morphological evidence and broader geographic sampling [23,27].

Because the present phylogeny is based on concatenated mitochondrial PCGs and does not represent a consensus species tree integrating mitochondrial and nuclear markers, branch-length patterns, voucher-based morphology, type-specimen comparisons, and broader geographic sampling, it is premature to synonymize *H. eigenmanni* with *H. leucisculus* solely on the basis of mitochondrial affinity [16,36,39,40]. In addition, the available dataset does not fully represent the geographic and taxonomic diversity of Hemiculter, and incomplete taxon sampling may affect both branch placement and distance-based interpretation. Therefore, the discordant positions of public records should be interpreted as evidence of mitogenomic inconsistency within the current reference dataset, not as definitive proof of species misidentification or taxonomic status. These results refine the taxonomic issue raised in previous *Hemiculter* studies [2,10]. Therefore, future taxonomic reassessment should test whether the observed pattern reflects synonymy, misidentification, introgression, or lineage-level divergence by combining complete mitogenomes with nuclear genomic data, branch-length-aware phylogenetic analyses, voucher-based morphological examination, and broader sampling across Korean, Chinese, Mongolian, and Amur River populations [23,27,28,40].

The barcode-gap analysis reinforces this cautious taxonomic interpretation [16,36]. Nominal intra- and interspecific K2P distances overlapped across most mitochondrial PCGs, but this overlap should not be interpreted as evidence that *Hemiculter* lacks informative barcode genes. Rather, it appears to result mainly from discordance between nominal species assignments and mitogenomic relationships in some public records, which can inflate apparent intraspecific distances and obscure interspecific separation. Therefore, individual mitochondrial PCGs may still be informative, but their utility for species identification should be evaluated together with voucher identity, phylogenetic placement, and mitogenome-scale evidence [11,17,19,20].

RSCU analysis showed a general preference for A/T-ending codons, which is consistent with the AT-rich composition of mitochondrial PCGs [17,41]. The broader RSCU heatmap based on the concatenated 13 PCGs also indicated generally conserved codon usage profiles among the analyzed *Hemiculter* mitogenomes, although minor lineage-specific differences were observed. However, these differences should be interpreted cautiously. Codon usage can be influenced by nucleotide composition, gene length, annotation boundaries, and sampling design [17,41]. Therefore, RSCU variation should be regarded as supporting information for describing codon usage patterns rather than as a direct diagnostic marker for distinguishing *Hemiculter* lineages [17,36].

The tRNA structures of the three newly assembled mitogenomes were also generally conserved, although several tRNAs showed minor sequence or structural differences among lineages. Such variation is not unexpected because mitochondrial tRNAs are short and structurally constrained but can still accumulate substitutions in stems or loops [28,30]. The observed tRNA differences may contribute to the characterization of each mitogenome, but they should not be overinterpreted as functional divergence without additional structural or experimental evidence [30,31]. In this context, the tRNA results are best interpreted as descriptive mitogenomic features that complement the PCG, RSCU, and phylogenetic analyses.

The dN/dS analysis indicated that all mitochondrial PCGs were under purifying selection, with ω values substantially lower than 1, a pattern consistent with reduced nonsynonymous substitution rates and strong functional constraint rather than direct evidence of purifying selection by itself [34,35]. This result is expected for mitochondrial PCGs, because they encode core components of oxidative phosphorylation and are generally subject to strong functional constraints [42,43]. Although low ω values are commonly interpreted as being compatible with purifying selection, such an interpretation should be made cautiously because dN/dS patterns alone do not provide unambiguous evidence for the underlying selective mechanism [44,45]. However, low ω values should not be interpreted as direct evidence that these genes are suitable barcode markers [16,36]. Rather, low ω indicates reduced amino-acid-level change under functional constraint [43,46]. The usefulness of mitochondrial genes for species identification should be evaluated using nucleotide-level divergence, nominal intra- and interspecific distances, and barcode-gap performance [13,36].

Taken together, our results show that *Hemiculter* mitogenomes are structurally conserved but contain sufficient nucleotide variation to reveal lineage-level divergence and taxonomic inconsistency among public records [23,28]. The newly generated mitogenomes provide useful genetic resources for *Hemiculter*, including mitogenomic records of *H. leucisculus* from Korea, *H. leucisculus* from Mongolia, and *H. eigenmanni* from Korea. Future studies should include broader geographic sampling across Korean, Mongolian, Chinese, and Amur River lineages and should combine complete mitogenomes with nuclear genomic data and voucher-based morphological examination [39,40]. Such integrative approaches will be necessary to clarify species boundaries, resolve possible synonymy or misidentification, and better understand the evolutionary history of *Hemiculter* within Cultrinae [2,23].

## 4. Materials and Methods

### 4.1. Sample Collection, DNA Extraction, and Sequencing

**Figure 8:**
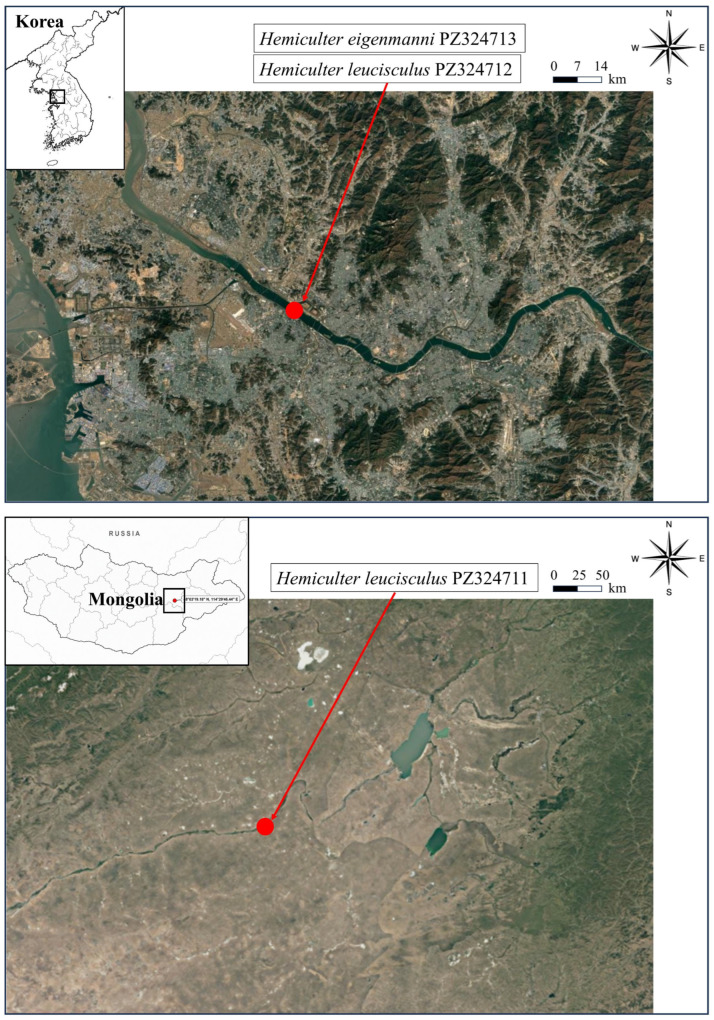
Specimens and sampling localities of the three newly sequenced *Hemiculter* lineages: *H. leucisculus* from Korea (PZ324712; Specimen No.: NNIBR-P31916), *H. leucisculus* from Mongolia (PZ324711; Specimen No.: NNIBR-P11594), and *H. eigenmanni* from Korea (PZ324713; Specimen No.: NNIBR-P31956). Sampling locations of *Hemiculter* specimens analyzed in this study. The (**upper**) panel shows the sampling locality in Korea, where *H. eigenmanni* PZ324713 and *H. leucisculus* PZ324712 were collected. The (**lower**) panel shows the sampling locality in Mongolia, where *H. leucisculus* PZ324711 was collected. Red circles indicate the sampling sites, and red lines connect each site to the corresponding species name and GenBank accession number. Inset maps indicate the geographic position of each sampling area within Korea and Mongolia, respectively. Scale bars and compass roses are shown in each panel. *H. leucisculus* from Mongolia was collected from the Kherlen River, Choibalsan, Mongolia. *H. leucisculus* from Korea and *H. eigenmanni* from Korea were collected from the Han River, Goyang-si, Republic of Korea.

*Hemiculter leucisculus* from Korea, *H. leucisculus* from Mongolia, and *H. eigenmanni* from Korea were sampled from three localities in 2020 using fishing nets; detailed information is provided in Figure 8 and Table S4. Pectoral fin tissues were preserved in 99% ethanol until DNA extraction. Genomic DNA was extracted from preserved fin tissues using the DNeasy Blood & Tissue Kit (QIAGEN, Germantown, MD, USA) according to the manufacturer’s instructions.

Specimens were identified morphologically before molecular analysis using standard taxonomic references for Korean freshwater fishes. Species identification followed Kim and Park [47] and Kim et al. [48], based on external diagnostic characters used for *Hemiculter* species, including overall body shape, mouth position, lateral-line scale pattern, fin position, and other meristic and morphological characters. The higher-level taxonomic classification followed Nelson [49]. Voucher specimens were deposited in the Nakdonggang National Institute of Biological Resources, and voucher numbers, sampling localities, coordinates, and GenBank accession numbers are provided in Figure 8 and Table S4.

The three *Hemiculter* mitogenomes were sequenced using 150 bp paired-end libraries prepared by Macrogen (Macrogen Inc., Seoul, Republic of Korea) on the Illumina HiSeq 2500 platform (Illumina, San Diego, CA, USA). All sampling procedures were conducted under the approval of the Nakdonggang National Institute of Biological Resources committee (approval no. 2023-04).

### 4.2. Sequence Assembly, Annotation, and Comparative Analysis

Illumina short-read data were filtered using Trimmomatic v0.39 [50] to remove adapter sequences and low-quality reads with quality scores below 20. Reads with more than 10% uncalled bases and duplicate reads were also removed. Filtered reads were used to assemble complete mitochondrial contigs using GetOrganelle v1.7.7.1 [51].

The total amount of raw and filtered sequencing data, Q30 values, estimated genome coverage, and mean assembly depth for each voucher specimen are summarized in Table S1. The assembled mitogenomes were annotated by comparison with available *Hemiculter* mitogenomes, and protein-coding genes (PCGs), tRNAs, and rRNAs were annotated using MITOS v2.1.10 [52]. tRNA genes and their predicted secondary structures were examined using tRNAscan-SE v2.0 [53]. For the three newly assembled mitogenomes, base composition and codon usage summaries were calculated using MEGA ver. 11.0 [54]. Nucleotide composition skewness was calculated using the following formulas: AT-skew = (A − T)/(A + T) and GC-skew = (G − C)/(G + C).

### 4.3. Sequence Variability, Genetic Distance, and Barcode-Gap Analyses

To quantitatively evaluate sequence divergence among *Hemiculter* mitogenomes, the 13 mitochondrial PCGs were extracted and aligned separately for each gene. The aligned PCGs were concatenated into a single 13-PCG dataset, and gene boundaries were retained for downstream gene-wise analyses. Pairwise genetic distances were calculated using the Kimura 2-parameter (K2P) model [55]. Gene-wise sequence variability was assessed by calculating the number of variable sites, parsimony-informative sites, singleton sites, and nucleotide diversity (Pi) for each PCG. Sliding-window analysis of nucleotide diversity was performed in R v.4.6.1 using a custom script based on the aligned concatenated 13-PCG dataset. The 13 PCG sequences were first aligned by gene and concatenated into a single alignment, and gene boundaries were recorded to annotate the sliding-window plot. Nucleotide diversity (Pi) was calculated for each 300 bp window with a 50 bp step size using the nuc.div function in the R package pegas v.1.4. Window-wise Pi values were plotted against the midpoint position of each window along the concatenated 13-PCG alignment using ggplot2 v4.0.0.

The mitochondrial control region was analyzed separately to evaluate length variation and sequence divergence. Control-region sequences were aligned, and aligned length, ungapped length, and gap counts were calculated for each sequence. Pairwise K2P distances were also estimated for the control-region alignment. Sliding-window nucleotide diversity of the control region was calculated using a window size of 100 bp and a step size of 20 bp.

To evaluate the discriminatory performance of individual mitochondrial PCGs, barcode-gap analyses were performed by comparing nominal intra- and interspecific K2P distances for each gene. For the broader *Hemiculter* mitogenome dataset, relative synonymous codon usage (RSCU) profiles based on concatenated 13 PCGs were calculated and visualized as a heatmap in R. All distance, diversity, barcode-gap, and RSCU analyses were conducted in R using the ape v5.8.1, pegas v1.4, seqinr v4.2.36, Biostrings v2.80.1, DECIPHER v3.8.0, tidyverse v2.0.0, ggplot2 v4.0.0, and pheatmap v1.0.13 packages.

### 4.4. Phylogenetic Analyses

Phylogenetic relationships were reconstructed using concatenated 13 PCGs from 18 mitogenomes representing *Hemiculter* and related Cultrinae taxa. Each of the 13 mitochondrial PCGs was aligned separately using MAFFT v7.453 [56] with default parameters, and the aligned genes were concatenated into a single dataset. FASTA files were converted to NEXUS and PHYLIP formats using Geneious v2023.11.15 [57].

To account for among-gene heterogeneity in substitution patterns and evolutionary rates, the concatenated 13-PCG alignment was partitioned by gene. Partitioned maximum-likelihood (ML) analysis was performed using IQ-TREE2 v.2.4.0 [58]. The best-fitting substitution models and partitioning scheme were selected using ModelFinder implemented in IQ-TREE2 [59], with partition merging allowed to reduce overparameterization. Branch support for the ML tree was assessed using 1000 ultrafast bootstrap replicates and 1000 SH-aLRT replicates [60,61].

Bayesian inference (BI) analysis was performed using MrBayes v3.2.7 [62]. BI analysis was performed using four concurrent Markov chain Monte Carlo chains for 5,000,000 generations, with sampling every 1000 generations and a burn-in of 25%. Convergence of the BI analysis was assessed by confirming that the average standard deviation of split frequencies decreased below 0.01 and that potential scale reduction factor values approached 1.0. The Bayesian inference analysis showed adequate convergence, with an average standard deviation of split frequencies of 0.006336, PSRF values close to 1.0 (0.999867–1.002079), and a minimum ESS of 1364.195, indicating reliable posterior sampling. Phylogenetic trees generated from both ML and BI analyses were visualized in FigTree v1.4.4 [63], and node support values were presented as BI posterior probabilities and ML bootstrap values. The phylogenetic tree was rooted using *Hypophthalmichthys nobilis* NC_010194 as the outgroup species.

### 4.5. Analysis of Selective Pressure on Mitochondrial PCGs of Cultrinae Species

To evaluate selective constraints acting on mitochondrial PCGs, nonsynonymous and synonymous substitution ratios (dN/dS, ω) were estimated for 13 mitochondrial PCGs from 18 Cultrinae mitogenomes. Each PCG was aligned using a codon-based alignment approach in MEGA ver. 11.0 [54]. For each mitochondrial PCG, dN, dS, and ω values were estimated using codeml implemented in EasyCodeML v4.10.7 [46,64]. The mean ω value for each PCG was used to evaluate selective constraint across the Cultrinae mitogenome dataset.

## 5. Conclusions

This study provides three newly assembled complete mitochondrial genomes of *Hemiculter*, including *H. leucisculus* from Korea, *H. leucisculus* from Mongolia, and *H. eigenmanni* from Korea. Comparative mitogenomic analyses showed that these mitogenomes retain the conserved gene content and organization typical of teleost mitochondrial genomes, while nucleotide-level variation across the 13 mitochondrial protein-coding genes was sufficient to reveal lineage-level divergence. Pairwise genetic distances and phylogenetic analyses showed that the newly assembled Korean and Mongolian *H. leucisculus* mitogenomes formed a distinct clade, whereas some publicly available *H. leucisculus* records were placed closer to *H. eigenmanni*-related or *H. tchangi*-related lineages. These patterns indicate that sequences currently assigned to *H. leucisculus* do not form a single consensus mitochondrial lineage and that the previously proposed synonymy between *H. eigenmanni* and *H. leucisculus* remains taxonomically controversial. Barcode-gap analyses further suggested that the overlap between nominal intra- and interspecific distances is likely influenced by discordant species assignments in public records rather than by the absence of informative mitochondrial barcode genes. Overall, our findings demonstrate the value of complete mitogenome-scale comparisons for detecting lineage-level divergence and taxonomic inconsistency and highlight the need for integrative taxonomic reassessment using broader sampling, voucher-based morphology, and nuclear genomic evidence.

## Figures and Tables

**Table 1:** Brief summary of the mitochondrial genome features of three newly assembled *Hemiculter* mitogenomes.

Species	Accession No.	Mitogenome Length (bp)	Gene Content	Control Region
*H. leucisculus* from Korea	PZ324712	16,623	13 PCGs, 22 tRNAs, 2 rRNAs, control region, OL	938 bp
*H. leucisculus* from Mongolia	PZ324711	16,618	13 PCGs, 22 tRNAs, 2 rRNAs, control region, OL	933 bp
*H. eigenmanni* from Korea	PZ324713	16,745	13 PCGs, 22 tRNAs, 2 rRNAs, control region, OL	937 bp

**Table 2:** Gene-wise sequence variability among 13 mitochondrial protein-coding genes of *Hemiculter* mitogenomes.

Gene	Length (bp)	Variable Sites	Parsimony-Informative Sites	Singleton Sites	Pi
*ND2*	1047	188	174	14	0.08047
*ND1*	975	245	152	93	0.07846
*ND6*	522	96	81	15	0.07079
*ND5*	1836	393	245	148	0.06747
*ATP6*	684	75	64	11	0.04414
*ND4L*	297	26	25	1	0.03974
*CYTB*	1140	148	72	76	0.03507
*ND3*	351	40	20	20	0.03061
*COX1*	1551	193	55	138	0.02881
*ND4*	1380	131	88	43	0.02725
*COX2*	690	63	28	35	0.02437
*ATP8*	165	9	8	1	0.02112
*COX3*	786	59	31	28	0.02009

**Table 3:** Pairwise K2P genetic distances among *Hemiculter* mitogenomes based on concatenated 13 mitochondrial protein-coding genes.

Taxon	Hb NC_029831	Hl PZ324711	Hl PZ324712	Hl AP012110	Hl MZ520999	He PZ324713	Hl NC_022929	He NC_029388	Hb KT361083	Hl MZ521000	Ht NC_036740	Hl KF956522
Hb NC_029831	-											
Hl PZ324711	0.092203	-										
Hl PZ324712	0.092199	0.002809	-									
Hl AP012110	0.108469	0.081365	0.080043	-								
Hl MZ520999	0.108605	0.08117	0.080257	0.011079	-							
He PZ324713	0.076039	0.032159	0.031511	0.072161	0.072371	-						
Hl NC_022929	0.080538	0.054192	0.053518	0.064984	0.065091	0.02142	-					
He NC_029388	0.081325	0.053817	0.053143	0.064902	0.065207	0.021333	0.001491	-				
Hb KT361083	0.089341	0.061274	0.060788	0.069526	0.069234	0.030543	0.012738	0.01283	-			
Hl MZ521000	0.086069	0.057247	0.056958	0.066062	0.065733	0.027021	0.009281	0.009373	0.006427	-		
Ht NC_036740	0.085412	0.056038	0.055555	0.065316	0.065025	0.026083	0.008651	0.008742	0.006158	0.002196	-	
Hl KF956522	0.08592	0.056728	0.056245	0.065726	0.065434	0.026546	0.008831	0.008922	0.006158	0.002196	0.001403	-

Hl = *Hemiculter leucisculus*; He = *Hemiculter eigenmanni*; Hb = *Hemiculter bleekeri*; Ht = *Hemiculter tchangi*.

## Data Availability

The original contributions presented in this study are included in the article and Supplementary Materials. Further inquiries can be directed to the corresponding author.

## Supplementary Materials

The following supporting information can be downloaded at: https://www.mdpi.com/article/10.3390/ijms27146325/s1.
