# Supplementary material for: Characterization of Three Complete Mitochondrial Genomes of Hemiculter and Comparative Mitogenomic Analysis of Cultrinae Fishes
Source: Int J Mol Sci. 2026 Jul 16;27(14):6325. doi: 10.3390/ijms27146325 (PMC13410722; doi:10.3390/ijms27146325)
Supplement: Supplementary file 1 [file ijms-27-06325-s001.zip › ijms-4314921-supplementary.pdf]

Table S1. Sequencing data statistics, quality metrics, and estimated coverage for voucher specimens

Voucher number	NCBI accession number	Raw total (bp)	Raw reads	Raw Q30 (%)	Raw coverage	Filtered total (bp)	Filtered reads	Filtered Q30 (%)	Filtered coverage
P11594	PZ324711	11,462,577,006	75,911,106	89.18	20.5×	9,098,140,462	60,648,774	93.22	16.3×
P31916	PZ324712	15,909,980,308	105,364,108	90.75	28.5×	13,436,453,677	89,189,190	93.99	24.0×
P31956	PZ324713	14,414,361,246	95,459,346	90.04	25.8×	11,926,327,410	79,168,324	93.73	21.3×

Table S2. Summary of the mitochondrial genome organization of three newly assembled *Hemiculter* mitogenomes.

Feature	*H. leucisculus* from Korea PZ324712					*H. leucisculus* from Mongolia PZ324711					*H. eigenmanni* from Korea PZ324713					Direction
	Position start/end	Length (bp)	Codon Start/end	AT skew	GC skew	Position start/end	Length (bp)	Codon Start/end	AT skew	GC skew	Position start/end	Length (bp)	Codon Start/end	AT skew	GC skew	
trnK	1-76	76				1-76	76				1-76	76				−
COX2	77-767	691	ATG/T--	0.084	−0.241	77-767	691	ATG/T--	0.071	−0.231	77-767	691	ATG/T--	0.065	−0.227	−
trnD	854-781	74				854-781	74				854-781	74				−
trnS2	858-918	71				858-918	71				858-918	71				+
COX1	929-2479	1551	GTG/TAA	−0.027	−0.174	929-2479	1551	GTG/TAA	−0.029	−0.176	929-2479	1551	GTG/TAA	−0.030	−0.185	−
trnY	2481-2551	71				2481-2551	71				2481-2551	71				+
trnC	2554-2661	68				2554-2661	68				2553-2660	68				+
trnN	2653-2725	73				2653-2725	73				2653-2725	73				+
trnA	2727-2795	69				2727-2795	69				2727-2795	69				+
trnW	2798-2868	71				2798-2868	71				2798-2868	71				−
ND2	2867-3913	1047	ATG/TAG	0.050	−0.261	2867-3913	1047	ATG/TAG	0.050	−0.256	2867-3913	1047	ATG/TAG	0.129	−0.339	−

trnM	3914-3982	69				3914-3982	69				3914-3982	69				−
trnQ	3984-4054	71				3984-4054	71				3984-4054	71				+
trnI	4053-4124	72				4053-4124	72				4053-4124	72				−
ND1	4129-5103	975	ATG/TAA	0.012	−0.222	4129-5103	975	ATG/TAA	0.010	−0.223	4129-5103	975	ATG/TAA	0.064	−0.277	−
trnL2	5105-5180	76				5105-5180	76				5105-5180	76				−
rrnL	5205-6851	1647				5205-6850	1646				5205-6849	1645				−
trnV	6871-6942	72				6870-6941	72				6992-7063	72				−
rrnS	6945-7906	962				6944-7905	962				7066-8027	962				−
trnF	7907-7975	69				7906-7974	69				8028-8096	69				−
trnP	8913-8982	70				8908-8977	70				9035-9104	70				+
trnT	8982-9053	72				8977-9048	72				9104-9175	72				−
CYTB	9054-101	1141	ATG/T--	0.014	−0.272	9049-10189	1141	ATG/T--	0.016	−0.282	9176-10316	1141	ATG/T--	0.003	−0.291	−

	94															
trnE	10199-10267	69				10194-10262	69				10321-10389	69				+
ND6	10268-10789	522	ATG/TAG	−0.451	0.341	10263-10784	522	ATG/TAG	−0.439	0.323	10390-10911	522	ATG/TAA	−0.439	0.359	+
ND5	10786-12621	1836	ATG/TAA	0.086	−0.331	10781-12616	1836	ATG/TAA	0.077	−0.317	10908-12743	1836	ATG/TAG	0.068	−0.313	−
trnL1	12622-12694	73				12617-12689	73				12744-12816	73				−
trnS1	12696-12764	69				12691-12759	69				12818-12886	69				−
trnH	12765-12833	69				12760-12828	69				12887-12955	69				−
ND4	12837-14215	1379	ATG/TA-	0.010	−0.231	12832-14210	1379	ATG/TA-	0.017	−0.235	12959-14337	1379	ATG/TA-	0.045	−0.268	−
ND4L	14209-14505	297	ATG/TAA	0.020	−0.319	14204-14500	297	ATG/TAA	0.007	−0.306	14331-14627	297	ATG/TAA	0.019	−0.333	−
trnR	14506-14575	70				14501-14570	70				14628-14697	70				−
ND3	14574-14924	351	ATG/TAG	−0.016	−0.263	14569-14919	351	ATG/TAG	−0.005	−0.278	14696-15046	351	ATG/TAG	−0.015	−0.299	−
trnG	14925-14996	72				14920-14991	72				15047-15118	72				−

COX3	14996-15781	786	ATG/TAA	0.014	−0.249	14991-15776	786	ATG/TAA	0.007	−0.245	15118-15903	786	ATG/TAA	0.012	−0.241	–
ATP6	15781-16464	684	ATG/TAA	−0.005	−0.356	15776-16459	684	ATG/TAA	−0.018	−0.343	15903-16586	684	ATG/TAA	−0.003	−0.393	–
ATP8	16458-16622	165	ATG/TAG	0.208	−0.438	16453-16617	165	ATG/TAG	0.208	−0.475	16580-16744	165	ATG/TAG	0.260	−0.508	–
Control region	8097-9034	938				7978-8910	933				7976-8912	937				
Origin of L-strand replication (OL)	2621-2652	32				2625-2655	31				2622-2652	31				

Table S3. Length variation of the mitochondrial control region among Hemiculter mitogenomes.

Scientific name	GenBank accession	Location	Aligned length	Ungapped length	Gap count
Hemiculter leucisculus	NC_022929	-	937	930	7
Hemiculter leucisculus	AP012110	-	937	936	1
Hemiculter leucisculus	KF956522	-	937	936	1
Hemiculter leucisculus	MZ520999	-	937	935	2
Hemiculter leucisculus	MZ521000	-	937	937	0
Hemiculter eigenmanni	NC_029388	-	937	937	0
Hemiculter leucisculus	PZ324711	Mongolia	937	932	5
Hemiculter eigenmanni	PZ324713	Korea	937	936	1
Hemiculter bleekeri	KT361083	-	937	930	7
Hemiculter bleekeri	NC_029831	-	937	927	10
Hemiculter tchangi	NC_036740	-	937	936	1
Hemiculter leucisculus	PZ324712	Korea	937	937	0

Table S4. Sampling sites and number of individuals in the study

Scientific name	Location	River and location	GenBank accession No.	N	Location
Hemiculter leucisculus	Mongolia	Kherlen River, Choibalsan	PZ324711	1	48°03'19.18″ N, 114°29'46.44" E
Hemiculter leucisculus	Korea	Han River, Goyang-si	PZ324712	1	37°35'21.93″ N, 126°50'08.25" E
Hemiculter eigenmanni	Korea	Han River, Goyang-si	PZ324713	1	37°35'39.18″ N, 126°49'54.20" E

N: Number of samples.
